# The Top 100 Most Cited Articles on Anterolateral Ligament: A Bibliometric Analysis and Review

**DOI:** 10.1055/s-0044-1800939

**Published:** 2025-03-04

**Authors:** Camilo Partezani Helito, Valdiane Pereira de Araujo, Sergio Marinho de Gusmao Canuto, Pedro Baches Jorge, Vitor Barion Castro de Padua, Diego Ariel de Lima

**Affiliations:** 1 Grupo de Joelho, Instituto de Ortopedia e Traumatologia, Hospital das Clínicas (HCFMUSP), Faculdade de Medicina da Universidade de São Paulo, São Paulo, SP, Brasil; 2 Universidade Estadual de Montes Claros (UNIMONTES), Montes Claros, MG, Brasil; 3 Ortoclinica Hospital de Ortopedia, Maceió, AL, Brasil; 4 Santa Casa de Misericórdia de São Paulo, São Paulo, SP, Brasil; 5 Universidade de Marília (UNIMAR), Marília, SP, Brasil; 6 Universidade Federal Rural do Semiárido (UFERSA), Mossoró, RN, Brasil

**Keywords:** bibliometric analysis, knee, ligaments, articular, publications

## Abstract

**Objectives** To conduct a bibliometric analysis aimed at evaluating the 100 most frequently cited articles concerning the anterolateral ligament (ALL).

**Methods** A thorough search was executed using the Scopus database with the keyword “Anterolateral Ligament.” The analysis incorporated technical notes, systematic reviews focusing on clinical outcomes and/or complications, clinical studies, and foundational scientific articles (both cadaveric and biomechanical). Case reports or personal opinion articles were omitted from the review. The 100 articles with the highest citation counts were examined using the Bibliometrix R-package software, which evaluated total citations, study type, country of origin, journal of publication, affiliated institution, and most prolific authors.

**Results** The 100 articles accumulated 11,192 citations, ranging from 44 to 703 per article. Most articles (69) were published between 2015 and 2017, predominantly focusing on anatomical and biomechanical studies. The United States was the leading country of publication (29%), followed by the United Kingdom (15%), Brazil (14%), France (13%), and Belgium (8%). The top five journals were the American Journal of Sports Medicine (31%), Arthroscopy (17%), Knee Surgery, Sports Traumatology, Arthroscopy (17%), Arthroscopy Techniques (5%), and Skeletal Radiology (4%). The most prolific authors were Sonnery-Cottet (21), Helito (17), with Musahl, Saithna, and Thaunat each contributing 11 articles. Sonnery-Cottet had the highest citation count, with 2,421.

**Conclusion** The analysis reveals that research on the ALL is growing, with significant contributions in anatomy and biomechanics. However, further studies are needed to establish the best indications for reconstruction and optimal surgical techniques.

## Introduction

**Fig. 1:**
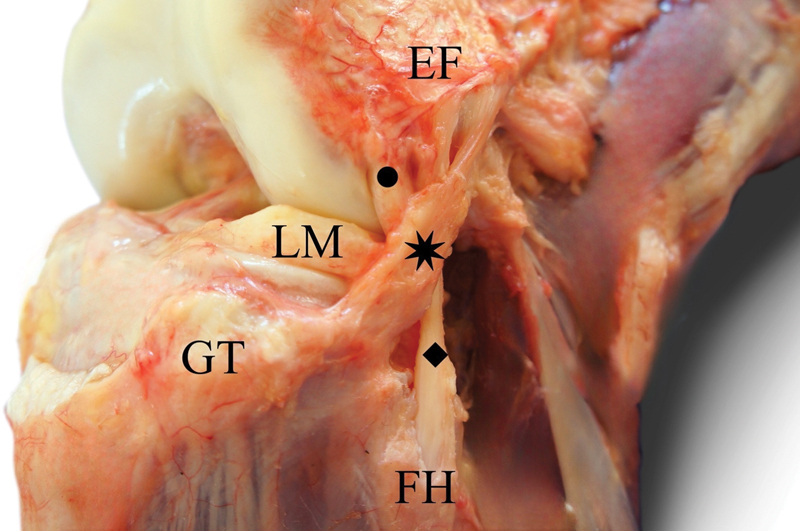
Anterolateral aspect of the knee, highlighting the relationship between the ALL, Lateral Collateral Ligament, and Popliteus Tendon. **Abbreviations:** FH, fibular head; GT, Gerdy tubercle; LM, lateral meniscus. Source: Ariel de Lima et al. 21

The anterolateral ligament (ALL) is a structure that has recently garnered significant attention due to its critical role in knee biomechanics, especially concerning anterolateral rotational stability. Positioned in the anterolateral region of the knee ( Fig. 1 ), the ALL is vital for rotational stability and contributes to limiting anterior tibial translation relative to the femur, albeit to a lesser extent. 1 Its importance becomes particularly evident in the context of anterior cruciate ligament (ACL) injuries, where rotational stability may remain compromised even after successful ACL reconstruction. 2 An injury to the ALL might contribute to the failure of ACL reconstruction, given its crucial role in maintaining knee rotational stability. Therefore, evaluating its integrity is essential in diagnosing and managing knee injuries, especially in patients who experience persistent rotational instability following ACL reconstruction. 3

Since the first detailed descriptions of the ALL by Helito et al., 4 Claes et al., 5 and Vincent et al. 6 in 2012 and 2013, there has been a notable increase in publications focusing on the ligament's anatomy, biomechanical characteristics, radiological markers, clinical outcomes, and complications related to its reconstruction. 1 Bibliometric analyses have emerged as valuable tools to delineate areas of consensus, identify controversial topics, explore research frontiers, and track current trends across various subjects, thereby providing readers with a comprehensive and up-to-date reference. A review of prior bibliometric studies indexed in the Scopus and PubMed databases revealed several analyses related to the reconstruction of tge anterior cruciate, 7 medial patellofemoral (MPFL), 8 and posterior cruciate (PCL) ligaments, 9 as well as of patellar instability. 10 However, to date, no bibliometric analysis has specifically addressed ALL.

Given the growing acceptance of ALL reconstruction as a standard procedure, this study was conducted to identify the 100 most cited articles published. The aim was to conduct a detailed bibliometric analysis aimed at evaluating the 100 most frequently cited articles concerning the ALL.

## Materials and Methods

### Data Collection and Allocation

A comprehensive search was performed using the Scopus database, which is recognized for its extensive repository of peer-reviewed scientific articles and detailed citation data, to support this literature review. The search utilized the term “Anterolateral Ligament” across all fields, with the results restricted to English-language publications and no limitations on publication dates. The search included technical notes, systematic reviews focused on clinical outcomes and/or complications, clinical studies, and foundational scientific articles (cadaveric and biomechanical). Case reports and personal opinions were excluded. To enhance accuracy, two of the authors independently selected articles for inclusion. The search was completed in July 2024, yielding 748 articles published since 2000.

**Fig. 2:**
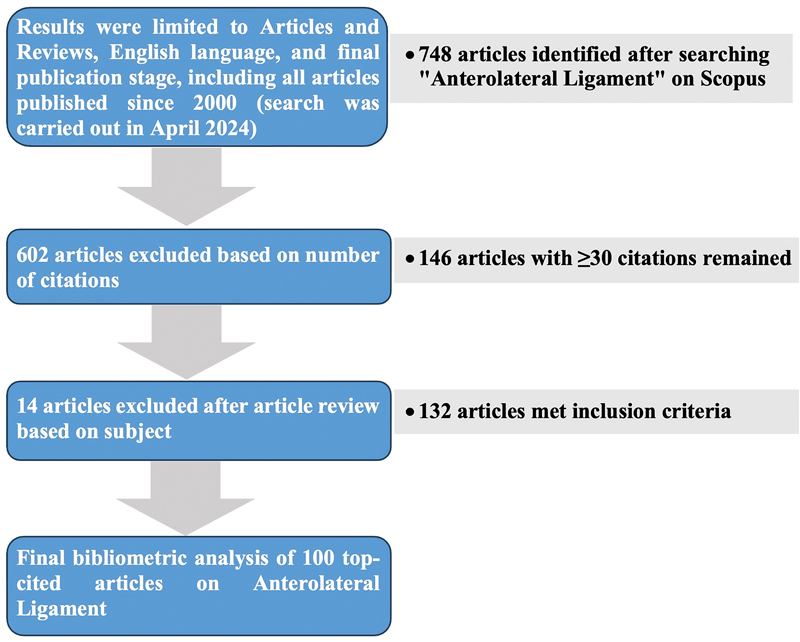
Flowchart illustrating the article selection procedure for the top 100 most cited articles on ALL.

These articles were ranked according to citation count, and after excluding those with fewer than 30 citations, 146 articles were retained for further analysis. Titles and abstracts were thoroughly reviewed, and each article was categorized into one of five study types: cadaveric (anatomical and/or biomechanical), clinical, computational/robotic, radiological, and systematic reviews. After excluding irrelevant articles and resolving any disagreements regarding inclusion, a total of 132 articles were retained. The articles defined as “irrelevant/controversial” were those with more than 30 citations but escaped the first filter of exclusion criteria (non-English language, case reports, and personal opinions). The top 100 most cited articles were then organized by citation count for the final analysis ( Fig. 2 ).

### Data Extraction

For data analysis, the Bibliometrix R-package software ( https://www.bibliometrix.org ) was utilized. In July 2024, a file with the “.bib” extension was extracted from Scopus. The full text of all selected articles was reviewed, and data were extracted, including title, author names, journal, year of publication, total number of citations, geographical origin, primary institution involved, and study type. Statistical analyses and data visualization were conducted using R and RStudio (R Foundation for Statistical Computing, Vienna, Austria), both of which are widely recognized open-source statistical software platforms.

## Results

The 100 most cited articles on the ALL are detailed in Table 1 . Research on this topic is relatively recent, as evidenced by the publication dates of the earliest articles in 2012 6 and 2013, 5 11 and the small total number of articles identified in our extensive search (748 results). In comparison, a PubMed search for “ACL” yielded over 38,000 results.

**Table 1:** The 100 most cited articles on the ALL

Rank	AU	PY	TI	SO	TC
**1**	Claes et al.	2013	Anatomy of the Anterolateral Ligament of the Knee	Journal of Anatomy	703
**2**	Sonnery-Cottet et al.	2015	Outcome of a Combined Anterior Cruciate Ligament and Anterolateral Ligament Reconstruction Technique with a Minimum 2-Year Follow-Up	American Journal of Sports Medicine	360
**3**	Dodds et al.	2014	The Anterolateral Ligament: Anatomy, Length Changes and Association with the Segond Fracture	Bone and Joint Journal	341
**4**	Vincent et al.	2012	The Anterolateral Ligament of the Human Knee: An Anatomic and Histologic Study	Knee Surgery, Sports Traumatology, Arthroscopy	337
**5**	Sonnery-Cottet et al.	2017	Anterolateral Ligament Reconstruction is Associated with Significantly Reduced ACL Graft Rupture Rates at a Minimum Follow-Up of 2 Years: A Prospective Comparative Study of 502 Patients from the Santi Study Group	American Journal of Sports Medicine	332
**6**	Kennedy et al.	2015	The Anterolateral Ligament: An Anatomic, Radiographic, and Biomechanical Analysis	American Journal of Sports Medicine	297
**7**	Kittl et al.	2016	The Role of the Anterolateral Structures and the ACL in Controlling Laxity of the Intact and ACL-Deficient Knee	American Journal of Sports Medicine	263
**8**	Parsons et al.	2015	The Biomechanical Function of the Anterolateral Ligament of the Knee	American Journal of Sports Medicine	257
**9**	Caterine et al.	2015	A Cadaveric Study of the Anterolateral Ligament: Reintroducing the Lateral Capsular Ligament	Knee Surgery, Sports Traumatology, Arthroscopy	246
**10**	Helito et al.	2013	Anatomy and Histology of the Knee Anterolateral Ligament	Orthopedic Journal of Sports Medicine	225
**11**	Getgood et al.	2019	The Anterolateral Complex of the Knee: Results from the International ALC Consensus Group Meeting	Knee Surgery, Sports Traumatology, Arthroscopy	224
**12**	Rasmussen et al.	2016	An In Vitro Robotic Assessment of the Anterolateral Ligament, Part 1	American Journal of Sports Medicine	218
**13**	Spencer et al.	2015	Biomechanical Analysis of Simulated Clinical Testing and Reconstruction of the Anterolateral Ligament of the Knee	American Journal of Sports Medicine	198
**14**	Inderhaug et al.	2017	Biomechanical Comparison of Anterolateral Procedures Combined with Anterior Cruciate Ligament Reconstruction	American Journal of Sports Medicine	195
**15**	Sonnery-Cottet et al.	2017	Anterolateral Ligament Expert Group Consensus Paper on the Management of Internal Rotation and Instability of the Anterior Cruciate Ligament – Deficient Knee	Journal of Orthopedics and Traumatology	188
**16**	Sonnery-Cottet et al.	2016	The Involvement of the Anterolateral Ligament in Rotational Cntrol of the Knee	American Journal of Sports Medicine	184
**17**	Claes et al.	2014	The Segond Fracture: A Bony Injury of the Anterolateral Ligament of the Knee	Arthroscopy – Journal of Arthroscopic and Related Surgery	169
**18**	Nitri et al.	2016	An In Vitro Robotic Assessment of the Anterolateral Ligament, Part 2	American Journal of Sports Medicine	166
**19**	Claes et al.	2014	High Prevalence of Anterolateral Ligament Abnormalities in Magnetic Resonance Images of Anterior Cruciate Ligament-Injured Knees	Acta Orthopaedica Belgica	154
**20**	Kittl et al.	2015	Length Change Patterns in the Lateral Extra-articular Structures of the Knee and Related Reconstructions	American Journal of Sports Medicine	153
**21**	Schon et al.	2016	Anatomic Anterolateral Ligament Reconstruction of the Knee Leads to Overconstraint at any Fixation Angle	American Journal of Sports Medicine	152
**22**	Ferretti et al.	2017	Prevalence and Classification of Injuries of Anterolateral Complex In Acute Anterior Cruciate Ligament Tears	Arthroscopy – Journal of Arthroscopic and Related Surgery	152
**23**	Musahl et al.	2016	The Influence of Meniscal and Anterolateral Capsular Injury on Knee Laxity in Patients with Anterior Cruciate Ligament Injuries	American Journal of Sports Medicine	146
**24**	Helito et al.	2014	MRI Evaluation of the Anterolateral Ligament of the Knee: Assessment in Routine 1.5-T Scans	Skeletal Radiology	137
**25**	Daggett et al.	2016	Femoral Origin of the Anterolateral Ligament: An Anatomic Analysis	Arthroscopy – Journal of Arthroscopic and Related Surgery	134
**26**	Geeslin et al.	2018	Anterolateral Knee Extra-Articular Stabilizers: A Robotic Study Comparing Anterolateral Ligament Reconstruction and Modified Lemaire Lateral Extra-Articular Tenodesis	American Journal of Sports Medicine	130
**27**	Inderhaug et al.	2017	Anterolateral Tenodesis or Anterolateral Ligament Complex Reconstruction: Effect of Flexion Angle at Graft Fixation When Combined with ACL Reconstruction	American Journal of Sports Medicine	126
**28**	Thein et al.	2016	Biomechanical Assessment of the Anterolateral Ligament of the Knee: A Secondary Restraint in Simulated Tests of the Pivot Shift and of Anterior Stability	Jurnal of Bone and Joint Surgery – American Volume	114
**29**	Porrino et al.	2015	The Anterolateral Ligament of the Knee: MRI Appearance, Association with the Segond Fracture, and Historical Perspective	American Journal of Roentgenology	113
**30**	Mackay et al.	2015	A Review of Ligament Augmentation with the Internalbrace™: The Surgical Principle is Described for the Lateral Ankle Ligament and ACL Repair in Particular, and a Comprehensive Review of Other Surgical Applications and Techniques is Presented	Surgical Technology International	103
**31**	Sonnery-Cottet et al.	2018	Epidemiological Evaluation of Meniscal Ramp Lesions in 3,214 Anterior Cruciate Ligament-Injured Knees from the Santi Study Group Database: A Rist Factor Analysis and Study of Secondary Meniscectomy Rates Following 769 Ramp Repairs	American Journal of Sports Medicine	100
**32**	Taneja et al.	2015	MRI Features of the Anterolateral Ligament of the Knee	Skeletal Radiology	100
**33**	Van Der Watt et al.	2015	The Structure and Function of the Anterolateral Ligament of the Knee: A Systematic Review	Arthroscopy – Journal of Arthroscopic and Related Surgery	99
**34**	Lee et al.	2019	Clinical Outcomes of Isolated Revision Anterior Cruciate Ligament Reconstruction or in Combination with Anatomic Anterolateral Ligament Reconstruction	American Journal of Sports Medicine	99
**35**	Helito et al.	2017	Assessment of the Anterolateral Ligament of the Knee by Magnetic Resonance Imaging in Acute Injuries of the Anterior Cruciate Ligament	Arthroscopy – Journal of Arthroscopic and Related Surgery	97
**36**	Helito et al.	2018	Combined Reconstruction of the Anterolateral Ligament in Chronic ACL Injuries Leads to Better Clinical Outcomes than Isolated ACL Reconstruction	Knee Surgery, Sports Traumatology, Arthroscopy	93
**37**	Helito et al.	2014	Radiographic Landmarks for Locating the Femoral Origin and Tibial Insertion of the Knee Anterolateral Ligament	American Journal of Sports Medicine	91
**38**	Ibrahim et al.	2017	Anatomic Reconstruction of the Anterior Cruciate Ligament of the Knee with or without Reconstruction of the Anterolateral Ligament: A Randomized Clinical Trial	American Journal of Sports Medicine	91
**39**	Sonnery-Cottet et al.	2018	Anterolateral Ligament Reconstruction Protects the Repaired Medial Meniscus: A Comparative Study of 383 Anterior Cruciate Ligament Reconstructions From The Santi Study Group with a Minimum Follow-Up of 2 Years	American Journal of Sports Medicine	90
**40**	Noyes et al.	2017	Is an Anterolateral Ligament Reconstruction Required in ACL-Reconstructed Knees With Associated Injury to the Anterolateral Structures? A Robotic Analysis of Rotational Knee Stability	American Journal of Sports Medicine	89
**41**	Van Dyck et al.	2016	Anterolateral Ligament Abnormalities in Patients with Acute Anterior Cruciate Ligament Rupture are Associated with Lateral Meniscal and Osseus Injuries	European Radiology	89
**42**	Helito et al.	2019	Combined Reconstruction of the Anterolateral Ligament in Patients with Anterior Cruciate Ligament Injury and Ligamentous Hyperlaxity Leads to Better Clinical Stability and a Lower Failure Rate than Isolated Anterior Cruciate Ligament Reconstruction	Arthroscopy – Journal of Arthroscopic and Related Surgery	87
**43**	Helito et al.	2015	Combined Intra- and Extra-Articular Reconstruction of the Anterior Cruciate Ligament: The Reconstruction of the Knee Anterolateral Ligament	Arthroscopy Techniques	86
**44**	Pomajzl et al.	2015	A Review of the Anterolateral Ligament of the Knee: Current Knowledge Regarding its Incidence, Anatomy, Biomechanics, and Surgical Dissection	Arthroscopy – Journal of Arthroscopic and Related Surgery	83
**45**	Thaunat et al.	2017	Reoperation Rates After Combined Anterior Cruciate Ligament and Anterolateral Ligament Reconstruction: A Series of 548 Patients from the Santi Study Group with a Minimum Follow-Up of 2 Years	American Journal of Sports Medicine	80
**46**	Kraeutler et al.	2018	Current Concepts of the Anterolateral Ligament of the Knee: Anatomy, Biomechanics, and Reconstruction	American Journal of Sports Medicine	79
**47**	Tavlo et al.	2016	The Role of the Anterolateral Ligament in ACL Insufficient and Reconstructed Knees on Rotatory Stability: A Biomechanical Study on Human Cadavers	Scandinavian Journal of Medicine & Science in Sports	79
**48**	Lutz et al.	2015	Behavior of the Anterolateral Structures of the Knee During Internal Rotation	Orthopedics and Traumatology: Surgery and Research	78
**49**	Stijak et al.	2016	Anatomic Description of the Anterolateral Ligament of the Knee	Knee Surgery, Sports Traumatology, Arthroscopy	77
**50**	Musahl et al.	2017	Contributions of the Anterolateral Complex and the Anterolateral Ligament to Rotatory Knee Stability in the Setting of ACL Injury: A Roundtable Discussion	Knee Surgery, Sports Traumatology, Arthroscopy	75
**51**	Smith et al.	2015	Combined Anterolateral Ligament and Anatomic Anterior Cruciate Ligament Reconstruction of the Knee	Knee Surgery, Sports Traumatology, Arthroscopy	75
**52**	Imbert et al.	2016	Isometric Characteristics of the Anterolateral Ligament of the Knee: A Cadaveric Navigation Study	Arthroscopy – Journal of Arthroscopic and Related Surgery	74
**53**	Sonnery-Cottet et al.	2016	Combined Anterior Cruciate Ligament and Anterolateral Ligament Reconstruction	Arthroscopy Techniques	74
**54**	Rahnemai-Azar et al.	2016	Structural Properties of the Anterolateral Capsule and Iliotibial Band of the Knee	American Journal of Sports Medicine	74
**55**	Noyes et al.	2017	Rotational Knee Instability in ACL-Deficient Knees: Role of the Anterolateral Ligament and Iliotibial Band as Defined by Tibiofemoral Compartment Translations and Rotations	Journal of Bone and Joint Surgery – American Volume	73
**56**	Zens et al.	2015	Length Changes of the Anterolateral Ligament During Passive Knee Motion	American Journal of Medicine	72
**57**	Dephillipo et al.	2017	Anterolateral Ligament Reconstruction Techniques, Biomechanics, and Clinical Outcomes: A Systematic Review	Arthroscopy – Journal of Arthroscopic and Related Surgery	71
**58**	Chahla et al.	2016	Anterolateral Ligament Reconstruction Technique: An Anatomic-Based Approach	Arthroscopy Techniques	70
**59**	Runer et al.	2016	The Anterolateral Ligament of the Knee: A Dissection Study	Knee	69
**60**	Grassi et al.	2020	Good Midterm Outcomes and Low Rates of Residual Rotatory Laxity, Complications and Failures After Revision Anterior Cruciate Ligament Reconstruction (ACL) and Lateral Extra-Articular Tenodesis (LET)	Knee Surgery, Sports Traumatology, Arthroscopy	69
**61**	Kosy et al.	2015	Characterization of the Anatomy of the Anterolateral Ligament of the Knee Using Magnetic Resonance Imaging	Skeletal Radiology	67
**62**	De Maeseneer et al.	2015	Segond Fracture: Involvement of the Iliotibial Band, Anterolateral Ligament, and Anterior Arm of the Biceps Femoris in Knee Trauma	Skeletal Radiology	67
**63**	Song et al.	2016	Bone Contusions After Acute Noncontact Anterior Cruciate Ligament Injury Are Associated With Knee Joint Laxity, Concomitant Meniscal Lesions, and Anterolateral Ligament Abnormality	Arthroscopy – Journal of Arthroscopic and Related Surgery	66
**64**	Saiegh et al.	2017	Sectioning the Anterolateral Ligament did not Increase Tibiofemoral Translation or Rotation in an ACL-Deficient Cadaveric Model	Knee Surgery, Sports Traumatology, Arthroscopy	66
**65**	Herbst et al.	2017	The Anterolateral Complex of the Knee: A Pictorial Essay	Knee Surgery, Sports Traumatology, Arthroscopy	65
**66**	Rosenstiel et al.	2019	Combined Anterior Cruciate and Anterolateral Ligament Reconstruction in the Professional Athlete: Clinicals Outcomes from the Scientific Anterior Cruciate Ligament Network International Study Group in a Series of 70 Patients with a Minimum Follow-Up of 2 Years	Arthroscopy – Journal of Arthroscopic and Related Surgery	64
**67**	Inderhaug et al.	2017	The Effects of Anterolateral Tenodesis on Tibiofemoral Contact Pressures and Kinematics	American Journal of Sports Medicine	64
**68**	Geeslin et al.	2018	Anterolateral Knee Extra-Articular Stabilizers: A Robotic Sectioning Study of the Anterolateral Ligament and Distal Iliotibial Band Kaplan Fibers	American Journal of Sports Medicine	63
**69**	Cavaignac et al.	2016	Ultrasonographic Identification of the Anterolateral Ligament of the Knee	Arthroscopy – Journal of Arthroscopic and Related Surgery	62
**70**	Aariel de Lima et al.	2019	Anatomy of the Anterolateral Ligament of the Knee: A Systematic Review	Arthroscopy – Journal of Arthroscopic and Related Surgery	62
**71**	Delaloye et al.	2020	Anterolateral Ligament Reconstruction and Modified Lemaire Lateral Extra-Articular Tenodesis Similarly Improve Knee Stability After Anterior Cruciate Ligament Reconstruction: A Biomechanical Study	Arthroscopy – Journal of Arthroscopic and Related Surgery	61
**72**	Williams et al.	2017	The Scientific Rationale for Lateral Tenodesis Augmentation of Intra-Articular ACL Reconstruction Using a Modified 'Lemaire' Procedure	Knee Surgery, Sports Traumatology, Arthroscopy	60
**73**	Cavaignac et al.	2017	Ultrasonographic Evaluation of Anterolateral Ligament Injuries: Correlation with Magnetic Resonance Imaging and Pivot-Shift Testing	Arthroscopy – Journal of Arthroscopic and Related Surgery	59
**74**	Sonnery-Cottet et al.	2021	Long-Term Graft Rupture Rates After Combined ACL and Anterolateral Ligament Reconstruction Versus Isolated ACL Reconstruction: A Matched-Pair Analysis from the Santi Study Group	American Journal of Sports Medicine	58
**75**	Marom et al.	2020	Lateral Extra-Articular Tenodesis Reduces Anterior Cruciate Ligament Graft Force and Anterior Tibial Translation in Response to Applied Pivoting and Anterior Drawer Loads	American Journal of Sports Medicine	58
**76**	Rezansoff et al.	2015	Radiographic Landmarks for Surgical Reconstruction of the Anterolateral Ligament of the Knee	Knee Surgery, Sports Traumatology, Arthroscopy	57
**77**	Saithna et al.	2018	Subspecialty Procedures: Combined ACL and Anterolateral Ligament Reconstruction	JBJS Essential Surgical Techniques	57
**78**	James et al.	2015	Anatomy and Biomechanics of the Lateral Side of the Knee and Surgical Implications	Sports Medicine and Arthroscopy Review	57
**79**	Musahl et al.	2016	Anterolateral Ligament of the Knee, Fact or Fiction?	Knee Surgery, Sports Traumatology, Arthroscopy	56
**80**	Sonnery-Cottet et al.	2016	Minimally Invasive Anterolateral Ligament Reconstruction in the Setting of Anterior Cruciate Ligament Injury	Arthroscopy Techniques	56
**81**	Daggett et al.	2016	Surgical Dissection of the Anterolateral Ligament	Arthroscopy Techniques	54
**82**	Dombrowski et al.	2016	Macroscopic Anatomical, Histological and Magnetic Resonance Imaging Correlation of the Lateral Capsule of the Knee	Knee Surgery, Sports Traumatology, Arthroscopy	53
**83**	Helito et al.	2015	Evaluation of the Anterolateral Ligament of the Knee by Means of Magnetic Resonance Examination [Avaliação do ligamento anterolateral do joelho por meio de exame de ressonância magnética]	Revista Brasileira de Ortopedia	53
**84**	Hartigan et al.	2016	Visibility of Anterolateral Ligament Tears in Anterior Cruciate Ligament-Deficient Knees with Standard 1.5-Tesla Magnetic Resonance Imaging	Arthroscopy – Journal of Arthroscopic and Related Surgery	52
**85**	Roessler et al.	2016	The Anterolateral Ligament (ALL) and its Role in Rotational Extra-Articular Stability of the Knee Joint: A Review of Anatomy and Surgical Concepts	Archives of Orthopedic and Trauma Surgery	52
**86**	Bonanzinga et al.	2017	Kinematics of ACL and Anterolateral Ligament. Part I: Combined Lesion	Knee Surgery, Sports Traumatology, Arthroscopy	51
**87**	Monaco et al.	2019	Correlation Between Magnetic Resonance Imaging and Surgical Exploration of the Anterolateral Structures of the Acute Anterior Cruciate Ligament-Injured Knee	American Journal of Sports Medicine	49
**88**	Wytrykowski et al.	2016	Cadaveric Study Comparing the Biomechanical Properties of Grafts Used for Knee Anterolateral Ligament Reconstruction	Arthroscopy – Journal of Arthroscopic and Related SurgeryY	48
**89**	Herbst et al.	2017	The Anterolateral Complex of the Knee	Orthopedic Journal of Sports Medicine	48
**90**	Helito et al.	2017	Anterolateral Ligament Abnormalities Are Associated with Peripheral Ligament and Osseous Injuries in Acute Ruptures of the Anterior Cruciate Ligament	Knee Surgery, Sports Traumatology, Arthroscopy	48
**91**	Thaunat et al.	2014	The Arcuate Ligament Revisited: Role of the Posterolateral Structures in Providing Static Stability in the Knee Joint	Knee Surgery, Sports Traumatology, Arthroscopy: Official Journal of the ESSKA	48
**92**	Zens et al.	2015	Mechanical Tensile Properties of the Anterolateral Ligament	Journal of Experimental Orthopedics	48
**93**	Sonnery-Cottet et al.	2018	Clinical Outcomes of Extra-Articular Tenodesis/Anterolateral Reconstruction in the ACL Injured Knee	Knee Surgery, Sports Traumatology, Arthroscopy	47
**94**	Lording et al.	2017	Rotational Laxity Control by the Anterolateral Ligament and the Lateral Meniscus is Dependent on Knee Flexion Angle: A Cadaveric Biomechanical Study	Clinical Orthopedics and Related Research	47
**95**	McDonald et al.	2017	Passive Anterior Tibial Subluxation in the Setting of Anterior Cruciate Ligament Injuries: A Comparative Analysis of Ligament-Deficient States	American Journal of Sports Medicine	46
**96**	Cianca et al.	2014	Musculoskeletal Ultrasound Imaging of the Recently Described Anterolateral Ligament of the Knee	American Journal of Physical Medicine and Rehabilitation	45
**97**	Helito et al.	2016	The Meniscal Insertion of the Knee Anterolateral Ligament	Surgical and Radiological Anatomy	45
**98**	Musahl et al.	2018	The Anterolateral Complex and Anterolateral Ligament of the Knee	Journal of the American Academy of Orthopedic Surgeons	45
**99**	Ferretti et al.	2019	High Prevalence of Anterolateral Ligament Abnormalities on MRI in Knees with Acute Anterior Cruciate Ligament Injuries: A Case-Control Series from the Santi Study Group	Orthopedic Journal of Sports Medicine	44
**100**	Thaunat et al.	2019	Hamstring Tendons or Bone-Patellar Tendon-Bone Graft for Anterior Cruciate Ligament Reconstruction?	Orthopedics and Traumatology: Surgery and Research	44

**Abbreviations:** AU, authors; PY, year of article publication; Rank, classification in descending order according to the number of citations; SO, journal; TC, total citations; TI, title.

**Fig. 3:**
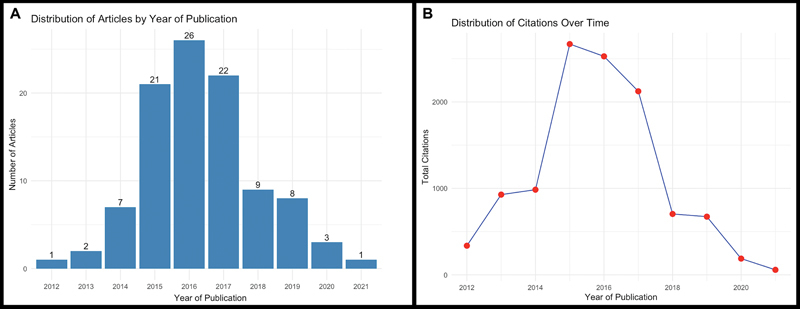
Distribution of the top 100 most cited articles on the ALL over the years. ( **A** ) All articles were published between 2012 and 2021. ( **B** ) Distribution of citations over the years.

The majority (69) of the top 100 articles were published between 2015 and 2017, as shown in Fig. 3 . The total number of citations for these articles was 11,192, with individual citations ranging from 44 to 703. The reduction in the number of top 100 most cited articles after 2017 suggests that citations are concentrated in these earlier publications. Despite an increase in recent ALL research, citations remain predominantly focused on earlier works. Just the first three articles, published in 2012 6 and 2013, 5 11 account for 1,265 citations (11.3%).

Categorizing the articles by study type (clinical, cadaveric, radiological, reviews, and computational studies), cadaveric studies accounted for 33% of the top 100 most cited articles. Of these, 12 were purely anatomical studies, while the remainder were biomechanical. Clinical studies were the second most common category (22%), followed by radiological studies (21%), literature reviews (18%), and computational studies (6%).

**Fig. 4:**
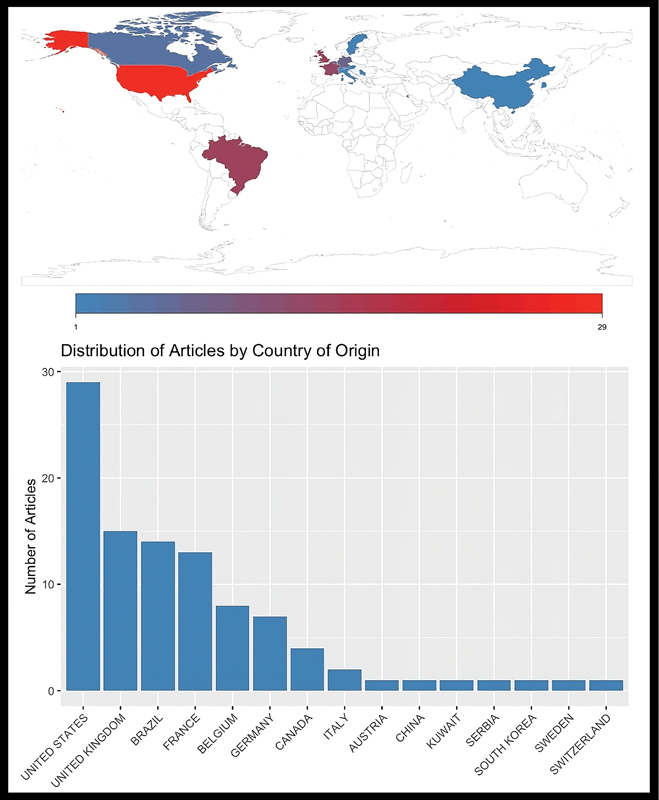
Distribution of the top 100 most cited articles on ALL according to the country of origin.

An analysis of the articles by country revealed that the United States was the most common country of publication (29%), followed by the United Kingdom (15%), Brazil (14%), France (13%), and Belgium (8%), as shown in Fig. 4 .

Institutions with the most cited articles among the top 100 on ALL include:

- Centre Orthopedique Paul Santy, Lyon, France;
- Institute of Orthopedics and Traumatology, University of São Paulo (IOT-HCFMUSP), São Paulo, Brazil;
- Steadman Philippon Research Institute, Vail, Colorado, United States;
- Department of Orthopaedic Surgery, University of Pittsburgh Medical Center, Pittsburgh, USA;
- Kansas City University, Kansas City, USA;
- Biomechanics Group, Mechanical Engineering Department, Imperial College London, United Kingdom;
- Ormskirk Hospital, Ormskirk, Lancashire, United Kingdom;
- Department of Orthopedic Surgery & Traumatology, University Hospitals Leuven, Leuven, Belgium;
- Orthopaedic Unit and Kirk Kilgour Sports Injury Center, Sant'Andrea University Hospital, “Sapienza” University of Rome, Rome, Italy;
- Hospital Sírio Libanês, São Paulo, Brazil.

Articles and citations per journal were also analyzed. Regarding the number of top 100 most cited articles, the five journals with the most top 100 published were: American Journal of Sports Medicine (31%), Arthroscopy – Journal of Arthroscopic and Related Surgery (17%), Knee Surgery, Sports Traumatology, Arthroscopy (17%), Arthroscopy Techniques (5%), and Skeletal Radiology (4%).

However, when we analyzed the journals by the number of citations, the order of the journals changed slightly: American Journal of Sports Medicine (4,380 citations), Knee Surgery, Sports Traumatology, Arthroscopy (1,699 citations), Arthroscopy – Journal of Arthroscopic and Related Surgery (1,440 citations), Journal of Anatomy (703 citations), and Skeletal Radiology (471 citations).

**Fig. 5:**
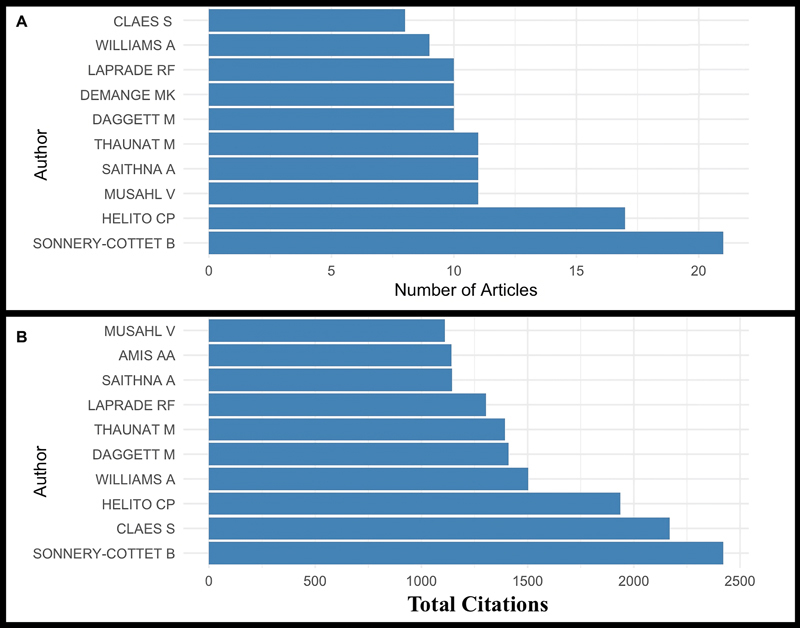
Distribution of the top 100 most cited articles on ALL. ( **A** ) Top 10 authors with the most articles on the list. ( **B** ) Top 10 authors with the most citations.

Regarding the authors of the top 100 most cited articles on ALL, in terms of the number of articles, we have Sonnery-Cottet (21), followed by Helito (17), and tied in third place with 11 each, Musahl, Saithna, and Thaunat. When considering the number of citations, we have Sonnery-Cottet with 2,421; Claes with 2,170; Helito with 1,935 citations; Williams with 1,501 citations; and closing the top 5 authors with the most citations, Daggett with 1,410 citations ( Fig. 5 ).

## Discussion

The research surrounding the ALL has considerable potential, especially in light of the significant advances made since 2012. The top 100 most cited articles on this topic have collectively received 11,192 citations, surpassing the 7,908 citations garnered by the top 50 most cited articles on the PCL since 1975. This count is approximately ⅓ of the 29,629 citations accumulated by the top cited articles on the ACL since 1975, being closely comparable to the 16,358 citations related to the MPFL. 8

The American Journal of Sports Medicine continues to be the leading journal in terms of the number of highly cited articles, consistent with findings from bibliometric studies on the ACL, PCL, and MPFL. The United States remains at the forefront of ALL research, with significant contributions also coming from the United Kingdom, Brazil, France, and Belgium.

An interesting case is that of the Journal of Anatomy, where its single entry on the list placed this journal among the top 5 most cited, that being Claes et al. 5 with 703 citations, which demonstrates how a single article can have a significant impact and relevance.

Regarding authorship, as depicted in Fig. 5 , the top 10 authors responsible for the most cited articles on the ALL collectively account for 118 publications. This reflects a high level of collaboration among these researchers, underscoring a strong cooperative environment, which is particularly notable given the relatively recent focus on this topic.

In reviewing the latest publications, we identified several key areas of controversy within ALL research, including measurements; trajectory and insertions; function and biomechanics; histology; vascularization; innervation; ultrasound; magnetic resonance imaging; combined reconstruction of the ALL and the ACL.

### Measurements of the ALL

Most studies report that the ALL measures between 33.0 and 42.0 mm in length, with this dimension increasing during knee flexion and internal rotation of the tibia. The ligament's width is typically found to be between 4.0 and 7.0 mm, while its thickness ranges from 1.0 to 2.0 mm. 1 4 Daggett et al., 12 in a comprehensive study involving over 160 specimens, observed that the average thickness of the ALL in men is approximately twice that observed in women.

### Trajectory and Insertions of the ALL

The ALL is characterized by three primary attachment points: femoral, tibial, and meniscal. It originates from the femur, near the lateral epicondyle, and follows an anteroinferior path toward the anterolateral aspect of the tibia. 1 The meniscal attachment is positioned between the body and the anterior horn of the lateral meniscus. Before connecting with the meniscus, the ALL fans out, increasing the insertion area. 13 14

The tibial attachment is located midway between the fibular head and the Gerdy tubercle, approximately 4.0 to 7.0 mm distal to the tibial plateau. 15

The femoral origin of the ALL shows the most variability among studies, with anatomical landmarks often including the lateral epicondyle of the femur and the origin of the lateral collateral ligament (LCL). Most studies describe the ALL as originating posterior and proximal to the lateral epicondyle. 1

### Function and Biomechanics of the ALL

The primary function of the ALL is to provide anterolateral stability to the knee, helping to prevent anterior and lateral subluxation of the tibia relative to the distal femur. 2 This stabilizing effect is partly attributed to the relatively posterior and proximal insertion of the ALL compared with the LCL. 16

### Histology of the ALL

Histological analysis of the ALL has revealed dense, well-organized collagen fibers, predominantly composed of type I collagen, with an average density of 121 fibroblasts/mm ^2^ in adults and 1,631 in fetuses, along with vascular tissue consistent with typical ligament morphology. 1

Macchi et al. 17 described the ALL as being primarily composed of type I collagen (90%), with smaller amounts of III (5%), VI (3%), and minimal elastic fibers (1%). The type I collagen was arranged in parallel, with wavy fibrils surrounded by type VI.

### Vascularization of the ALL

Situated within the third layer of the anterolateral aspect of the knee, the ALL is closely associated with the lateral inferior genicular vessels, which are separated by a thin layer of adipose tissue. 17 These vessels are situated between the ALL and the lateral meniscus, serving as an anatomical landmark for identification. 18 19

### Innervation of the ALL

Caterine et al. 20 identified the presence of neurofilament protein within the ALL, suggesting that it is innervated by peripheral nerves. Their research uncovered circular structures that likely correspond to small peripheral nerves or mechanoreceptors. Ariel de Lima et al., 21 through an immunofluorescence study using protein gene product 9.5, determined that this ligament contains peripheral nerve structures, primarily type I and IV mechanoreceptors. These findings imply that the ALL plays a significant role in proprioception and contributes to the anterolateral stabilization of the knee.

### Ultrasonography of the ALL

Cianca et al. 22 and both studies by Cavaignac et al., 23 24 utilizing ultrasonography, reported a 100% success rate in visualizing the ALL. Oshima et al. 25 found that most segments could be clearly identified, establishing ultrasonography as a valuable tool for diagnosing related injuries. Conversely, Capo et al., 26 who achieved a 75% visualization rate, noted that ultrasound was not consistently effective in reliably identifying the ligament's tibial and femoral origins.

### Magnetic Resonance Imaging of the ALL

The ALL can be visualized on conventional 1.5T magnetic resonance imaging (MRI) scans, particularly on coronal sections and T2-weighted images with fat saturation. The visualization rate can reach up to 97.8%, with the meniscal portion being the most frequently observed (94.8%). 19 Caterine et al., 20 in a study using 3.0T MRI on cadavers, achieved 100% visualization. However, in many cases, the femoral origin was not distinctly visible on coronal plane images, primarily due to the close association of the ALL with other ligamentous structures. Nevertheless, the tibial and meniscal insertions were identifiable.

Kosy et al. 27 and Helito et al., 28 in 1.5T MRI studies, reported good results in identifying the meniscal portion of the ALL. Taneja et al., 29 in contrast, in studies involving both 1.5 and 3.0T MRI, were unable to identify the meniscal insertion.

### Combined Reconstruction of the Anterolateral and Anterior Cruciate Ligaments

In a review study, Ariel de Lima et al., 3 concluded that the main surgical indications for combined reconstruction of the ACL and ALL are: revision surgery, a physical examination with pivot shift grade 2 or 3, participation in sports with a pivot mechanism and/or high levels of activity, ligamentous laxity, and presence of a Segond fracture. Secondary indications may include those aged under 25 years, chronic ACL injury, and radiological signs of lateral femoral condyle depression.

## Limitations

Our research included only articles published in English, which means that high-quality studies in other languages may have been overlooked. Furthermore, data collection was limited to the Scopus database, as it is known for its accuracy and extensive coverage, and it allowed us to export citation data; other databases, such as PubMed or the Cochrane Library, were not included in our search. Additionally, our research was restricted to the ligament specifically described as “Anterolateral,” potentially excluding relevant studies that refer to it by other names, such as the “Lateral Capsular Ligament” or the “Anterior Oblique Band.”

Another limitation arises from the nature of bibliometric research itself, as the articles included may not fully represent the current standard of care and evidence. Despite this, the bibliometric analysis of the top 100 most cited articles on ALL offers a valuable foundation for identifying research trends and highlighting key research centers. It also provides a curated list of influential articles, serving as essential reading material for researchers and new residents aiming to build upon the existing body of literature on this subject.

## Conclusion

This analysis reveals that research on ALL is growing, with significant contributions in anatomy and biomechanics. However, further studies are needed to establish the best indications for ALL reconstruction and optimal surgical techniques.
