# Supplementary material for: The Top 100 Most Cited Articles on Anterolateral Ligament: A Bibliometric Analysis and Review
Source: Rev Bras Ortop (Sao Paulo). 2025 Mar 4;60(1):s00441800939. doi: 10.1055/s-0044-1800939 (PMC11879490; doi:10.1055/s-0044-1800939)
Supplement: Related texts

# Research article: Os 100 artigos mais citados sobre o ligamento anterolateral: Uma análise bibliométrica e revisão

**Keywords:** análise bibliométrica, joelho, ligamentos, articulares, publicações

## Abstract

**Objetivos** Realizar uma análise bibliométrica visando avaliar os 100 artigos mais citados sobre o ligamento anterolateral (LAL).

**Métodos** Uma busca completa foi executada usando o banco de dados Scopus com a palavra-chave *anterolateral ligament* (ligamento anterolateral). A análise incorporou notas técnicas, revisões sistemáticas com foco em desfechos clínicos e/ou complicações, estudos clínicos e artigos científicos fundamentais (cadavéricos e biomecânicos). Relatos de caso ou opiniões pessoais foram omitidos da revisão. Os 100 artigos com os maiores números de citações foram examinados com o programa Bibliometrix R-package, que avaliou citações totais, tipo de estudo, país de origem, periódico de publicação, instituição afiliada e autores mais prolíficos.

**Resultados** Os 100 artigos acumularam 11.192 citações, variando de 44 a 703 por artigo. A maioria dos artigos (69) foi publicada entre 2015 e 2017, com foco predominante em estudos anatômicos e biomecânicos. Os Estados Unidos foram o principal país de publicação (29%), seguidos pelo Reino Unido (15%), Brasil (14%), França (13%) e Bélgica (8%). Os cinco principais periódicos foram o *American Journal of Sports Medicine* (31%), *Arthroscopy* (17%), *Knee Surgery, Sports Traumatology, Arthroscopy* (17%), *Arthroscopy Techniques* (5%) e *Skeletal Radiology* (4%). Os autores mais prolíficos foram Sonnery-Cottet (21), Helito (17) e Musahl, Saithna e Thaunat contribuíram com 11 artigos cada. Sonnery-Cottet teve o maior número de citações, com 2.421.

**Conclusão** A análise revela que a pesquisa sobre o LAL está crescendo, com contribuições significativas em anatomia e biomecânica. No entanto, estudos adicionais são necessários para estabelecer as melhores indicações para a reconstrução e as técnicas cirúrgicas ideais.

## Introdução

O ligamento anterolateral (LAL) é uma estrutura que recentemente ganhou atenção significativa devido ao seu papel crítico na biomecânica do joelho, especialmente no que diz respeito à estabilidade rotacional anterolateral. Posicionado na região anterolateral do joelho ( Fig. 1 ), o LAL é vital para a estabilidade rotacional e contribui para limitar a translação anterior da tíbia em relação ao fêmur, embora em menor grau. 1 Sua importância é particularmente evidente no contexto de lesões do ligamento cruzado anterior (LCA), em que a estabilidade rotacional pode continuar comprometida mesmo após a reconstrução bem-sucedida do LCA. 2 Uma lesão no LAL pode contribuir para a falha da reconstrução do LCA devido ao seu papel crucial na manutenção da estabilidade rotacional do joelho. Portanto, a avaliação de sua integridade é essencial no diagnóstico e tratamento de lesões no joelho, especialmente em pacientes que apresentam instabilidade rotacional persistente após a reconstrução do LCA. 3

**Fig. 1:** Aspecto anterolateral do joelho, destacando a relação entre o Ligamento Anterolateral, o Ligamento Colateral Lateral e o Tendão Poplíteo. **Notas:**  Ligamento Anterolateral;  Ligamento Colateral Lateral; ● Tendão Poplíteo. **Abreviações:** CF, cabeça fibular; TG, tubérculo de Gerdy; ML, menisco lateral. Nota: Imagem gentilmente cedida por Ariel de Lima et al. 21

Desde as primeiras descrições detalhadas do LAL por Helito et al., 4 Claes et al. 5 e Vincent et al. 6 em 2012 e 2013, houve um aumento notável nas publicações com foco na anatomia do ligamento, características biomecânicas, marcadores radiológicos, desfechos clínicos e complicações relacionadas à sua reconstrução. 1 As análises bibliométricas surgiram como ferramentas valiosas para delinear áreas de consenso, identificar tópicos controversos, explorar fronteiras de pesquisa e rastrear tendências atuais em vários assuntos, dando aos leitores uma referência abrangente e atualizada. Uma revisão de estudos bibliométricos anteriores indexados nas bases de dados Scopus e PubMed revelou várias análises relacionadas à reconstrução dos ligamentos cruzado anterior, 7 patelofemoral medial (LPFM), 8 cruzado posterior (LCP), 9 assim como da instabilidade patelar. 10 No entanto, até o momento, não há análises bibliométricas especificamente sobre o LAL.

Dada a crescente aceitação da reconstrução do LAL como procedimento padrão, este estudo foi conduzido para identificar os 100 artigos mais citados já publicados. O objetivo foi realizar uma análise bibliométrica detalhada visando avaliar os 100 artigos mais citados sobre o LAL.

## Materiais e Métodos

## Coleta de Dados e Alocação

Uma busca abrangente foi realizada usando o banco de dados Scopus, que é reconhecido por seu extenso repositório de artigos científicos revistos por pares e dados detalhados de citações para dar suporte a esta revisão de literatura. A busca utilizou o termo *anterolateral ligament* (ligamento anterolateral”) em todos os campos, com os resultados restritos a publicações em inglês e sem limitações nas datas de publicação. A busca incluiu notas técnicas, revisões sistemáticas focadas em desfechos clínicos e/ou complicações, estudos clínicos e artigos científicos fundamentais (cadavéricos e biomecânicos). Relatos de casos e opiniões pessoais foram excluídos. Para aumentar a precisão, dois dos autores selecionaram artigos independentemente para inclusão. A busca, concluída em julho de 2024, gerou 748 artigos publicados desde 2000.

Esses artigos foram classificados de acordo com o número de citações e, após excluir aqueles com menos de 30 citações, 146 artigos foram retidos para análise posterior. Títulos e resumos foram revisados minuciosamente e cada artigo foi categorizado em um dos cinco tipos de estudo: cadavérico (anatômico e/ou biomecânico), clínico, computacional/robótico, radiológico e revisões sistemáticas. Após excluir artigos irrelevantes e resolver quaisquer divergências quanto à inclusão, um total de 132 artigos foram mantidos. Os artigos definidos como “irrelevantes/controversos” foram aqueles com mais de 30 citações, mas que escaparam do primeiro filtro de critérios de exclusão (idioma além do inglês, relatos de caso e opiniões pessoais). Os 100 artigos mais citados foram, então, organizados por número de citações para a análise final ( Fig. 2 ).

**Fig. 2:** Fluxograma ilustrando o procedimento de seleção de artigos para os 100 artigos mais citados sobre o Ligamento Anterolateral.

## Extração de Dados

A análise de dados utilizou o programa Bibliometrix R-package ( https://www.bibliometrix.org ). Em julho de 2024, um arquivo com a extensão “.bib” foi extraído do Scopus. O texto completo de todos os artigos selecionados foi revisado e os dados foram extraídos, incluindo título, nomes dos autores, periódico, ano de publicação, número total de citações, origem geográfica, instituição primária envolvida e tipo de estudo. Análises estatísticas e visualização de dados foram conduzidas usando R e RStudio (R Foundation for Statistical Computing, Viena, Áustria), duas plataformas de *software* estatístico de código aberto amplamente conhecidas.

## Resultados

A Tabela 1 mostra os 100 artigos mais citados sobre o LAL. Pesquisas nesse tópico são relativamente recentes, como mostram as datas de publicação dos primeiros artigos, em 2012 6 e 2013, 5 11 e o pequeno número total de artigos identificados em nossa extensa busca (748 resultados). Em comparação, uma busca no PubMed por *LCA* gerou mais de 38.000 resultados.

**Tabela 1:** Os artigos mais citados sobre o ligamento anterolateral

Rank	AU	PY	TI	SO	TC
**1**	Claes et al.	2013	Anatomy of the Anterolateral Ligament of the Knee	Journal of Anatomy	703
**2**	Sonnery-Cottet et al.	2015	Outcome of a Combined Anterior Cruciate Ligament and Anterolateral Ligament Reconstruction Technique with a Minimum 2-Year Follow-Up	American Journal of Sports Medicine	360
**3**	Dodds et al.	2014	The Anterolateral Ligament: Anatomy, Length Changes and Association with the Segond Fracture	Bone and Joint Journal	341
**4**	Vincent et al.	2012	The Anterolateral Ligament of the Human Knee: An Anatomic and Histologic Study	Knee Surgery, Sports Traumatology, Arthroscopy	337
**5**	Sonnery-Cottet et al.	2017	Anterolateral Ligament Reconstruction is Associated with Significantly Reduced ACL Graft Rupture Rates at a Minimum Follow-Up of 2 Years: A Prospective Comparative Study of 502 Patients from the Santi Study Group	American Journal of Sports Medicine	332
**6**	Kennedy et al.	2015	The Anterolateral Ligament: An Anatomic, Radiographic, and Biomechanical Analysis	American Journal of Sports Medicine	297
**7**	Kittl et al.	2016	The Role of the Anterolateral Structures and the ACL in Controlling Laxity of the Intact and ACL-Deficient Knee	American Journal of Sports Medicine	263
**8**	Parsons et al.	2015	The Biomechanical Function of the Anterolateral Ligament of the Knee	American Journal of Sports Medicine	257
**9**	Caterine et al.	2015	A Cadaveric Study of the Anterolateral Ligament: Reintroducing the Lateral Capsular Ligament	Knee Surgery, Sports Traumatology, Arthroscopy	246
**10**	Helito et al.	2013	Anatomy and Histology of the Knee Anterolateral Ligament	Orthopedic Journal of Sports Medicine	225
**11**	Getgood et al.	2019	The Anterolateral Complex of the Knee: Results from the International ALC Consensus Group Meeting	Knee Surgery, Sports Traumatology, Arthroscopy	224
**12**	Rasmussen et al.	2016	An In Vitro Robotic Assessment of the Anterolateral Ligament, Part 1	American Journal of Sports Medicine	218
**13**	Spencer et al.	2015	Biomechanical Analysis of Simulated Clinical Testing and Reconstruction of the Anterolateral Ligament of the Knee	American Journal of Sports Medicine	198
**14**	Inderhaug et al.	2017	Biomechanical Comparison of Anterolateral Procedures Combined with Anterior Cruciate Ligament Reconstruction	American Journal of Sports Medicine	195
**15**	Sonnery-Cottet et al.	2017	Anterolateral Ligament Expert Group Consensus Paper on the Management of Internal Rotation and Instability of the Anterior Cruciate Ligament – Deficient Knee	Journal of Orthopedics and Traumatology	188
**16**	Sonnery-Cottet et al.	2016	The Involvement of the Anterolateral Ligament in Rotational Cntrol of the Knee	American Journal of Sports Medicine	184
**17**	Claes et al.	2014	The Segond Fracture: A Bony Injury of the Anterolateral Ligament of the Knee	Arthroscopy – Journal of Arthroscopic and Related Surgery	169
**18**	Nitri et al.	2016	An In Vitro Robotic Assessment of the Anterolateral Ligament, Part 2	American Journal of Sports Medicine	166
**19**	Claes et al.	2014	High Prevalence of Anterolateral Ligament Abnormalities in Magnetic Resonance Images of Anterior Cruciate Ligament-Injured Knees	Acta Orthopaedica Belgica	154
**20**	Kittl et al.	2015	Length Change Patterns in the Lateral Extra-articular Structures of the Knee and Related Reconstructions	American Journal of Sports Medicine	153
**21**	Schon et al.	2016	Anatomic Anterolateral Ligament Reconstruction of the Knee Leads to Overconstraint at any Fixation Angle	American Journal of Sports Medicine	152
**22**	Ferretti et al.	2017	Prevalence and Classification of Injuries of Anterolateral Complex In Acute Anterior Cruciate Ligament Tears	Arthroscopy – Journal of Arthroscopic and Related Surgery	152
**23**	Musahl et al.	2016	The Influence of Meniscal and Anterolateral Capsular Injury on Knee Laxity in Patients with Anterior Cruciate Ligament Injuries	American Journal of Sports Medicine	146
**24**	Helito et al.	2014	MRI Evaluation of the Anterolateral Ligament of the Knee: Assessment in Routine 1.5-T Scans	Skeletal Radiology	137
**25**	Daggett et al.	2016	Femoral Origin of the Anterolateral Ligament: An Anatomic Analysis	Arthroscopy – Journal of Arthroscopic and Related Surgery	134
**26**	Geeslin et al.	2018	Anterolateral Knee Extra-Articular Stabilizers: A Robotic Study Comparing Anterolateral Ligament Reconstruction and Modified Lemaire Lateral Extra-Articular Tenodesis	American Journal of Sports Medicine	130
**27**	Inderhaug et al.	2017	Anterolateral Tenodesis or Anterolateral Ligament Complex Reconstruction: Effect of Flexion Angle at Graft Fixation When Combined with ACL Reconstruction	American Journal of Sports Medicine	126
**28**	Thein et al.	2016	Biomechanical Assessment of the Anterolateral Ligament of the Knee: A Secondary Restraint in Simulated Tests of the Pivot Shift and of Anterior Stability	Jurnal of Bone and Joint Surgery – American Volume	114
**29**	Porrino et al.	2015	The Anterolateral Ligament of the Knee: MRI Appearance, Association with the Segond Fracture, and Historical Perspective	American Journal of Roentgenology	113
**30**	Mackay et al.	2015	A Review of Ligament Augmentation with the Internalbrace™: The Surgical Principle is Described for the Lateral Ankle Ligament and ACL Repair in Particular, and a Comprehensive Review of Other Surgical Applications and Techniques is Presented	Surgical Technology International	103
**31**	Sonnery-Cottet et al.	2018	Epidemiological Evaluation of Meniscal Ramp Lesions in 3,214 Anterior Cruciate Ligament-Injured Knees from the Santi Study Group Database: A Rist Factor Analysis and Study of Secondary Meniscectomy Rates Following 769 Ramp Repairs	American Journal of Sports Medicine	100
**32**	Taneja et al.	2015	MRI Features of the Anterolateral Ligament of the Knee	Skeletal Radiology	100
**33**	Van Der Watt et al.	2015	The Structure and Function of the Anterolateral Ligament of the Knee: A Systematic Review	Arthroscopy – Journal of Arthroscopic and Related Surgery	99
**34**	Lee et al.	2019	Clinical Outcomes of Isolated Revision Anterior Cruciate Ligament Reconstruction or in Combination with Anatomic Anterolateral Ligament Reconstruction	American Journal of Sports Medicine	99
**35**	Helito et al.	2017	Assessment of the Anterolateral Ligament of the Knee by Magnetic Resonance Imaging in Acute Injuries of the Anterior Cruciate Ligament	Arthroscopy – Journal of Arthroscopic and Related Surgery	97
**36**	Helito et al.	2018	Combined Reconstruction of the Anterolateral Ligament in Chronic ACL Injuries Leads to Better Clinical Outcomes than Isolated ACL Reconstruction	Knee Surgery, Sports Traumatology, Arthroscopy	93
**37**	Helito et al.	2014	Radiographic Landmarks for Locating the Femoral Origin and Tibial Insertion of the Knee Anterolateral Ligament	American Journal of Sports Medicine	91
**38**	Ibrahim et al.	2017	Anatomic Reconstruction of the Anterior Cruciate Ligament of the Knee with or without Reconstruction of the Anterolateral Ligament: A Randomized Clinical Trial	American Journal of Sports Medicine	91
**39**	Sonnery-Cottet et al.	2018	Anterolateral Ligament Reconstruction Protects the Repaired Medial Meniscus: A Comparative Study of 383 Anterior Cruciate Ligament Reconstructions From The Santi Study Group with a Minimum Follow-Up of 2 Years	American Journal of Sports Medicine	90
**40**	Noyes et al.	2017	Is an Anterolateral Ligament Reconstruction Required in ACL-Reconstructed Knees With Associated Injury to the Anterolateral Structures? A Robotic Analysis of Rotational Knee Stability	American Journal of Sports Medicine	89
**41**	Van Dyck et al.	2016	Anterolateral Ligament Abnormalities in Patients with Acute Anterior Cruciate Ligament Rupture are Associated with Lateral Meniscal and Osseus Injuries	European Radiology	89
**42**	Helito et al.	2019	Combined Reconstruction of the Anterolateral Ligament in Patients with Anterior Cruciate Ligament Injury and Ligamentous Hyperlaxity Leads to Better Clinical Stability and a Lower Failure Rate than Isolated Anterior Cruciate Ligament Reconstruction	Arthroscopy – Journal of Arthroscopic and Related Surgery	87
**43**	Helito et al.	2015	Combined Intra- and Extra-Articular Reconstruction of the Anterior Cruciate Ligament: The Reconstruction of the Knee Anterolateral Ligament	Arthroscopy Techniques	86
**44**	Pomajzl et al.	2015	A Review of the Anterolateral Ligament of the Knee: Current Knowledge Regarding its Incidence, Anatomy, Biomechanics, and Surgical Dissection	Arthroscopy – Journal of Arthroscopic and Related Surgery	83
**45**	Thaunat et al.	2017	Reoperation Rates After Combined Anterior Cruciate Ligament and Anterolateral Ligament Reconstruction: A Series of 548 Patients from the Santi Study Group with a Minimum Follow-Up of 2 Years	American Journal of Sports Medicine	80
**46**	Kraeutler et al.	2018	Current Concepts of the Anterolateral Ligament of the Knee: Anatomy, Biomechanics, and Reconstruction	American Journal of Sports Medicine	79
**47**	Tavlo et al.	2016	The Role of the Anterolateral Ligament in ACL Insufficient and Reconstructed Knees on Rotatory Stability: A Biomechanical Study on Human Cadavers	Scandinavian Journal of Medicine & Science in Sports	79
**48**	Lutz et al.	2015	Behavior of the Anterolateral Structures of the Knee During Internal Rotation	Orthopedics and Traumatology: Surgery and Research	78
**49**	Stijak et al.	2016	Anatomic Description of the Anterolateral Ligament of the Knee	Knee Surgery, Sports Traumatology, Arthroscopy	77
**50**	Musahl et al.	2017	Contributions of the Anterolateral Complex and the Anterolateral Ligament to Rotatory Knee Stability in the Setting of ACL Injury: A Roundtable Discussion	Knee Surgery, Sports Traumatology, Arthroscopy	75
**51**	Smith et al.	2015	Combined Anterolateral Ligament and Anatomic Anterior Cruciate Ligament Reconstruction of the Knee	Knee Surgery, Sports Traumatology, Arthroscopy	75
**52**	Imbert et al.	2016	Isometric Characteristics of the Anterolateral Ligament of the Knee: A Cadaveric Navigation Study	Arthroscopy – Journal of Arthroscopic and Related Surgery	74
**53**	Sonnery-Cottet et al.	2016	Combined Anterior Cruciate Ligament and Anterolateral Ligament Reconstruction	Arthroscopy Techniques	74
**54**	Rahnemai-Azar et al.	2016	Structural Properties of the Anterolateral Capsule and Iliotibial Band of the Knee	American Journal of Sports Medicine	74
**55**	Noyes et al.	2017	Rotational Knee Instability in ACL-Deficient Knees: Role of the Anterolateral Ligament and Iliotibial Band as Defined by Tibiofemoral Compartment Translations and Rotations	Journal of Bone and Joint Surgery – American Volume	73
**56**	Zens et al.	2015	Length Changes of the Anterolateral Ligament During Passive Knee Motion	American Journal of Medicine	72
**57**	Dephillipo et al.	2017	Anterolateral Ligament Reconstruction Techniques, Biomechanics, and Clinical Outcomes: A Systematic Review	Arthroscopy – Journal of Arthroscopic and Related Surgery	71
**58**	Chahla et al.	2016	Anterolateral Ligament Reconstruction Technique: An Anatomic-Based Approach	Arthroscopy Techniques	70
**59**	Runer et al.	2016	The Anterolateral Ligament of the Knee: A Dissection Study	Knee	69
**60**	Grassi et al.	2020	Good Midterm Outcomes and Low Rates of Residual Rotatory Laxity, Complications and Failures After Revision Anterior Cruciate Ligament Reconstruction (ACL) and Lateral Extra-Articular Tenodesis (LET)	Knee Surgery, Sports Traumatology, Arthroscopy	69
**61**	Kosy et al.	2015	Characterization of the Anatomy of the Anterolateral Ligament of the Knee Using Magnetic Resonance Imaging	Skeletal Radiology	67
**62**	De Maeseneer et al.	2015	Segond Fracture: Involvement of the Iliotibial Band, Anterolateral Ligament, and Anterior Arm of the Biceps Femoris in Knee Trauma	Skeletal Radiology	67
**63**	Song et al.	2016	Bone Contusions After Acute Noncontact Anterior Cruciate Ligament Injury Are Associated With Knee Joint Laxity, Concomitant Meniscal Lesions, and Anterolateral Ligament Abnormality	Arthroscopy – Journal of Arthroscopic and Related Surgery	66
**64**	Saiegh et al.	2017	Sectioning the Anterolateral Ligament did not Increase Tibiofemoral Translation or Rotation in an ACL-Deficient Cadaveric Model	Knee Surgery, Sports Traumatology, Arthroscopy	66
**65**	Herbst et al.	2017	The Anterolateral Complex of the Knee: A Pictorial Essay	Knee Surgery, Sports Traumatology, Arthroscopy	65
**66**	Rosenstiel et al.	2019	Combined Anterior Cruciate and Anterolateral Ligament Reconstruction in the Professional Athlete: Clinicals Outcomes from the Scientific Anterior Cruciate Ligament Network International Study Group in a Series of 70 Patients with a Minimum Follow-Up of 2 Years	Arthroscopy – Journal of Arthroscopic and Related Surgery	64
**67**	Inderhaug et al.	2017	The Effects of Anterolateral Tenodesis on Tibiofemoral Contact Pressures and Kinematics	American Journal of Sports Medicine	64
**68**	Geeslin et al.	2018	Anterolateral Knee Extra-Articular Stabilizers: A Robotic Sectioning Study of the Anterolateral Ligament and Distal Iliotibial Band Kaplan Fibers	American Journal of Sports Medicine	63
**69**	Cavaignac et al.	2016	Ultrasonographic Identification of the Anterolateral Ligament of the Knee	Arthroscopy – Journal of Arthroscopic and Related Surgery	62
**70**	Aariel de Lima et al.	2019	Anatomy of the Anterolateral Ligament of the Knee: A Systematic Review	Arthroscopy – Journal of Arthroscopic and Related Surgery	62
**71**	Delaloye et al.	2020	Anterolateral Ligament Reconstruction and Modified Lemaire Lateral Extra-Articular Tenodesis Similarly Improve Knee Stability After Anterior Cruciate Ligament Reconstruction: A Biomechanical Study	Arthroscopy – Journal of Arthroscopic and Related Surgery	61
**72**	Williams et al.	2017	The Scientific Rationale for Lateral Tenodesis Augmentation of Intra-Articular ACL Reconstruction Using a Modified 'Lemaire' Procedure	Knee Surgery, Sports Traumatology, Arthroscopy	60
**73**	Cavaignac et al.	2017	Ultrasonographic Evaluation of Anterolateral Ligament Injuries: Correlation with Magnetic Resonance Imaging and Pivot-Shift Testing	Arthroscopy – Journal of Arthroscopic and Related Surgery	59
**74**	Sonnery-Cottet et al.	2021	Long-Term Graft Rupture Rates After Combined ACL and Anterolateral Ligament Reconstruction Versus Isolated ACL Reconstruction: A Matched-Pair Analysis from the Santi Study Group	American Journal of Sports Medicine	58
**75**	Marom et al.	2020	Lateral Extra-Articular Tenodesis Reduces Anterior Cruciate Ligament Graft Force and Anterior Tibial Translation in Response to Applied Pivoting and Anterior Drawer Loads	American Journal of Sports Medicine	58
**76**	Rezansoff et al.	2015	Radiographic Landmarks for Surgical Reconstruction of the Anterolateral Ligament of the Knee	Knee Surgery, Sports Traumatology, Arthroscopy	57
**77**	Saithna et al.	2018	Subspecialty Procedures: Combined ACL and Anterolateral Ligament Reconstruction	JBJS Essential Surgical Techniques	57
**78**	James et al.	2015	Anatomy and Biomechanics of the Lateral Side of the Knee and Surgical Implications	Sports Medicine and Arthroscopy Review	57
**79**	Musahl et al.	2016	Anterolateral Ligament of the Knee, Fact or Fiction?	Knee Surgery, Sports Traumatology, Arthroscopy	56
**80**	Sonnery-Cottet et al.	2016	Minimally Invasive Anterolateral Ligament Reconstruction in the Setting of Anterior Cruciate Ligament Injury	Arthroscopy Techniques	56
**81**	Daggett et al.	2016	Surgical Dissection of the Anterolateral Ligament	Arthroscopy Techniques	54
**82**	Dombrowski et al.	2016	Macroscopic Anatomical, Histological and Magnetic Resonance Imaging Correlation of the Lateral Capsule of the Knee	Knee Surgery, Sports Traumatology, Arthroscopy	53
**83**	Helito et al.	2015	Evaluation of the Anterolateral Ligament of the Knee by Means of Magnetic Resonance Examination [Avaliação do ligamento anterolateral do joelho por meio de exame de ressonância magnética]	Revista Brasileira de Ortopedia	53
**84**	Hartigan et al.	2016	Visibility of Anterolateral Ligament Tears in Anterior Cruciate Ligament-Deficient Knees with Standard 1.5-Tesla Magnetic Resonance Imaging	Arthroscopy – Journal of Arthroscopic and Related Surgery	52
**85**	Roessler et al.	2016	The Anterolateral Ligament (ALL) and its Role in Rotational Extra-Articular Stability of the Knee Joint: A Review of Anatomy and Surgical Concepts	Archives of Orthopedic and Trauma Surgery	52
**86**	Bonanzinga et al.	2017	Kinematics of ACL and Anterolateral Ligament. Part I: Combined Lesion	Knee Surgery, Sports Traumatology, Arthroscopy	51
**87**	Monaco et al.	2019	Correlation Between Magnetic Resonance Imaging and Surgical Exploration of the Anterolateral Structures of the Acute Anterior Cruciate Ligament-Injured Knee	American Journal of Sports Medicine	49
**88**	Wytrykowski et al.	2016	Cadaveric Study Comparing the Biomechanical Properties of Grafts Used for Knee Anterolateral Ligament Reconstruction	Arthroscopy – Journal of Arthroscopic and Related SurgeryY	48
**89**	Herbst et al.	2017	The Anterolateral Complex of the Knee	Orthopedic Journal of Sports Medicine	48
**90**	Helito et al.	2017	Anterolateral Ligament Abnormalities Are Associated with Peripheral Ligament and Osseous Injuries in Acute Ruptures of the Anterior Cruciate Ligament	Knee Surgery, Sports Traumatology, Arthroscopy	48
**91**	Thaunat et al.	2014	The Arcuate Ligament Revisited: Role of the Posterolateral Structures in Providing Static Stability in the Knee Joint	Knee Surgery, Sports Traumatology, Arthroscopy: Official Journal of the ESSKA	48
**92**	Zens et al.	2015	Mechanical Tensile Properties of the Anterolateral Ligament	Journal of Experimental Orthopedics	48
**93**	Sonnery-Cottet et al.	2018	Clinical Outcomes of Extra-Articular Tenodesis/Anterolateral Reconstruction in the ACL Injured Knee	Knee Surgery, Sports Traumatology, Arthroscopy	47
**94**	Lording et al.	2017	Rotational Laxity Control by the Anterolateral Ligament and the Lateral Meniscus is Dependent on Knee Flexion Angle: A Cadaveric Biomechanical Study	Clinical Orthopedics and Related Research	47
**95**	McDonald et al.	2017	Passive Anterior Tibial Subluxation in the Setting of Anterior Cruciate Ligament Injuries: A Comparative Analysis of Ligament-Deficient States	American Journal of Sports Medicine	46
**96**	Cianca et al.	2014	Musculoskeletal Ultrasound Imaging of the Recently Described Anterolateral Ligament of the Knee	American Journal of Physical Medicine and Rehabilitation	45
**97**	Helito et al.	2016	The Meniscal Insertion of the Knee Anterolateral Ligament	Surgical and Radiological Anatomy	45
**98**	Musahl et al.	2018	The Anterolateral Complex and Anterolateral Ligament of the Knee	Journal of the American Academy of Orthopedic Surgeons	45
**99**	Ferretti et al.	2019	High Prevalence of Anterolateral Ligament Abnormalities on MRI in Knees with Acute Anterior Cruciate Ligament Injuries: A Case-Control Series from the Santi Study Group	Orthopedic Journal of Sports Medicine	44
**100**	Thaunat et al.	2019	Hamstring Tendons or Bone-Patellar Tendon-Bone Graft for Anterior Cruciate Ligament Reconstruction?	Orthopedics and Traumatology: Surgery and Research	44

**Abreviações:** AP, ano de publicação; classificação, classificação em ordem descendente de acordo com o número de citações; TC, total de citações.

A maioria dos 100 principais artigos (69) foi publicada entre 2015 e 2017, como mostra a Fig. 3 . O número total de citações desses artigos foi de 11.192, com citações individuais variando de 44 a 703. A redução no número dos 100 artigos mais citados após 2017 sugere que as citações estão concentradas nessas publicações anteriores. Apesar de um aumento na pesquisa recente sobre o LAL, as citações permanecem predominantemente focadas em trabalhos anteriores. Apenas os três primeiros artigos, publicados em 2012 6 e 2013, 5 11 foram responsáveis por 1.265 citações (11,3%).

**Fig. 3:** Distribuição dos 100 artigos mais citados sobre o ligamento anterolateral (LAL) ao longo dos anos. ( **A** ) Todos os artigos mais citados foram publicados entre 2012 e 2021. ( **B** ) Distribuição de citações dos artigos mais citados sobre LAL ao longo dos anos.

Na categorização dos artigos por tipo de estudo (clínico, cadavérico, radiológico, revisões e estudos computacionais), os estudos cadavéricos foram responsáveis por 33% dos 100 artigos mais citados. Destes, 12 eram estudos puramente anatômicos e os restantes eram biomecânicos. Estudos clínicos foram a segunda categoria mais comum (22%), seguidos por estudos radiológicos (21%), revisões de literatura (18%) e estudos computacionais (6%).

Uma análise dos artigos por país revelou que os Estados Unidos foram o primeiro país de publicação (29%), seguidos por Reino Unido (15%), Brasil (14%), França (13%) e Bélgica (8%), como notado na Fig. 4 .

**Fig. 4:** Distribuição dos 100 artigos mais citados sobre o ligamento anterolateral (LAL) por país de origem.

As instituições com os 100 artigos mais citados sobre LAL são:

- Centre Orthopedique Paul Santy, Lyon, França;
- Instituto de Ortopedia e Traumatologia, Universidade de São Paulo (IOT-HCFMUSP), São Paulo, Brasil;
- Steadman Philippon Research Institute, Vail, Colorado, Estados Unidos;
- Departamento de Cirurgia Ortopédica, University of Pittsburgh Medical Center, Pittsburgh, Estados Unidos;
- Kansas City University, Kansas City, Estados Unidos;
- Grupo de Biomecânica, Departamento de Engenharia Mecânica, Imperial College London, Londres, Reino Unido;
- Ormskirk Hospital, Ormskirk, Lancashire, Reino Unido;
- Departamento de Cirurgia Ortopédica e Traumatologia, University Hospitals Leuven, Leuven, Bélgica;
- Unidade Ortopédica e Centro Kirk Kilgour de Lesões Desportivas, Sant'Andrea University Hospital, “Sapienza” University of Rome, Roma, Itália;
- Hospital Sírio Libanês, São Paulo, Brasil.

Artigos e citações por periódico também foram analisados. Em relação ao número dos 100 artigos mais citados, os cinco periódicos com mais publicações foram o *American Journal of Sports Medicine* (31%), *Arthroscopy – Journal of Arthroscopic and Related Surgery* (17%), *Knee Surgery, Sports Traumatology, Arthroscopy* (17%), *Arthroscopy Techniques* (5%) e *Skeletal Radiology* (4%).

No entanto, quando analisamos os periódicos pelo número de citações, a ordem dos periódicos mudou ligeiramente para *American Journal of Sports Medicine* (4.380 citações), *Knee Surgery, Sports Traumatology, Arthroscopy* (1.699 citações), *Arthroscopy - Journal of Arthroscopic and Related Surgery* (1.440 citações), *Journal of Anatomy* (703 citações) e *Skeletal Radiology* (471 citações).

Em relação aos autores dos 100 artigos mais citados sobre LAL, em termos de número de artigos, temos Sonnery-Cottet (21), seguido por Helito (17) e, empatados em terceiro lugar, com 11 artigos entre os 100 principais cada, Musahl, Saithna e Thaunat. Ao considerar o número de citações, temos Sonnery-Cottet com 2.421, Claes com 2.170, Helito com 1.935, Williams com 1.501 e, por fim, Daggett com 1.410 citações ( Fig. 5 ).

**Fig. 5:** Distribuição dos 100 artigos mais citados sobre o ligamento anterolateral. ( **A** ) Dez principais autores com os artigos mais citados. ( **B** ) Dez principais autores com mais citações.

## Discussão

A pesquisa sobre o LAL tem potencial considerável, especialmente à luz dos avanços significativos feitos desde 2012. Os 100 artigos mais citados nesse tema receberam coletivamente 11.192 citações, superando as 7.908 citações obtidas pelos 50 artigos mais citados sobre o LCP desde 1975. Esse número é aproximadamente ⅓ das 29.629 citações acumuladas pelos 100 artigos mais citados sobre o LCA desde 1975 e é bastante comparável às 16.358 citações relacionadas ao LPFM. 8

O *American Journal of Sports Medicine* continua a ser o periódico líder em termos de número de artigos altamente citados, consistente com os achados de estudos bibliométricos sobre LCA, LCP e LPFM. Os Estados Unidos permanecem na vanguarda da pesquisa de LAL, com contribuições significativas também vindas do Reino Unido, Brasil, França e Bélgica.

Um caso interessante é o *Journal of Anatomy* , já que seu único artigo dentre os 100 principais o colocou dentre os cinco maiores, sendo ele por Claes et al. 5 com 703 citações, demonstrando como uma única publicação pode ter impacto e relevância significativos.

Quanto à autoria, como mostrado na Fig. 5 , os dez principais autores responsáveis pelos artigos mais citados sobre o LAL coletivamente fizeram 118 publicações. Isso reflete um alto nível de colaboração entre esses pesquisadores, ressaltando um forte ambiente cooperativo, o que é bastante notável dado o foco relativamente recente nesse tópico.

Ao revisar as publicações mais recentes, identificamos diversas áreas-chave de controvérsia na pesquisa da LAL, incluindo medidas; trajetória e inserções; função e biomecânica; histologia; vascularização; inervação; ultrassom; ressonância magnética; e reconstrução combinada do LAL e do LCA.

## Medidas do LAL

A maioria dos estudos relata que o LAL tem entre 33,0 e 42,0 mm de comprimento, aumentando durante a flexão do joelho e a rotação interna da tíbia. A largura do ligamento é tipicamente de 4,0 a 7,0 mm, enquanto sua espessura varia de 1,0 a 2,0 mm. 1 4 Daggett et al., 12 em um estudo abrangente com mais de 160 espécimes, observaram que a espessura média do LAL em homens é aproximadamente o dobro da observada em mulheres.

## Trajetória e Inserções do LAL

O LAL é caracterizado por três pontos de fixação primários: femoral, tibial e meniscal. Este ligamento se origina do fêmur, próximo ao epicôndilo lateral, e segue uma trajetória anteroinferior em direção ao aspecto anterolateral da tíbia. 1 A inserção meniscal fica entre o corpo e o corno anterior do menisco lateral. Antes de se conectar com o menisco, o LAL se abre em leque, aumentando a área de inserção. 13 14

A inserção tibial está localizada a meio caminho entre a cabeça da fíbula e o tubérculo de Gerdy, cerca de 4,0 a 7,0 mm distal ao platô tibial. 15

A origem femoral do LAL mostra a maior variabilidade entre os estudos e os marcos anatômicos frequentemente incluem o epicôndilo lateral do fêmur e a origem do ligamento colateral lateral (LCL). A maioria dos estudos descreve o LAL com origem posterior e proximal ao epicôndilo lateral. 1

## Função e Biomecânica do LAL

A função primária do LAL é dar estabilidade anterolateral ao joelho, ajudando a prevenir a subluxação anterior e lateral da tíbia em relação ao fêmur distal. 2 Esse efeito estabilizador é, em parte, atribuído à inserção relativamente posterior e proximal do LAL em comparação ao LCL. 16

## Histologia do LAL

A análise histológica do LAL revelou fibras colágenas densas e bem-organizadas, compostas predominantemente por colágeno de tipo I, com densidade média de 121 fibroblastos/mm ^2^ em adultos e 1.631 em fetos, juntamente com tecido vascular condizente com a morfologia típica do ligamento. 1

Macchi et al. 17 descreveram o LAL como composto principalmente por colágeno de tipo I (90%), com quantidades menores de III (5%), VI (3%) e fibras elásticas mínimas (1%). O colágeno de tipo I foi organizado em paralelo, com fibrilas onduladas cercadas por tipo VI.

## Vascularização do LAL

Situado na terceira camada do aspecto anterolateral do joelho, o LAL está intimamente associado aos vasos geniculares inferiores laterais, que são separados por uma fina camada de tecido adiposo. 17 Esses vasos estão situados entre o LAL e o menisco lateral, servindo como um marco anatômico para identificação. 18 19

## Inervação do LAL

Caterine et al. 20 identificaram a presença de proteína neurofilamento dentro do LAL, sugerindo que sua inervação é feita por nervos periféricos. Sua pesquisa revelou estruturas circulares que provavelmente correspondem a pequenos nervos periféricos ou mecanorreceptores. Ariel de Lima et al., 21 por meio de um estudo de imunofluorescência usando a proteína produto do gene 9.5, determinaram que o esse ligamento contém estruturas nervosas periféricas, principalmente mecanorreceptores dos tipos I e IV. Estes achados implicam que o LAL desempenha um papel significativo na propriocepção e contribui para a estabilização anterolateral do joelho.

## Ultrassonografia do LAL

Cianca et al. 22 e os dois estudos de Cavaignac et al., 23 24 utilizando ultrassonografia, relataram uma taxa de sucesso de 100% na visualização do LAL. Oshima et al. 25 descobriram que a maioria dos segmentos poderia ser claramente identificada, estabelecendo a ultrassonografia como uma ferramenta valiosa para o diagnóstico de lesões deste ligamento. Por outro lado, Capo et al., 26 que atingiram uma taxa de visualização de 75%, observaram que o ultrassom não foi consistentemente eficaz na identificação confiável das origens tibial e femoral do ligamento.

## Ressonância Magnética do LAL

O LAL pode ser visualizado em exames de ressonância magnética (RM) convencionais de 1,5 T, particularmente em cortes coronais e imagens ponderadas em T2 com saturação de gordura. A taxa de visualização pode chegar a 97,8%, sendo a porção meniscal a mais observada (94,8%). 19 Caterine et al., 20 em um estudo usando RM de 3,0 T em cadáveres, obtiveram 100% de visualização. No entanto, em muitos casos, a origem femoral não era distintamente visível em imagens do plano coronal, em especial devido à associação próxima do LAL com outras estruturas ligamentares. Ainda assim, as inserções tibial e meniscal eram identificáveis.

Kosy et al. 27 e Helito et al., 28 em estudos de RM de 1,5 T, relataram bons resultados na identificação da porção meniscal do LAL. Taneja et al., 29 por outro lado, em estudos com RM de 1,5 e 3,0 T, não foram capazes de identificar a inserção meniscal.

## Reconstrução Combinada do Ligamento Anterolateral e do Ligamento Cruzado Anterior

Em um estudo de revisão, Ariel de Lima et al. 3 concluíram que as principais indicações cirúrgicas para reconstrução combinada do LCA e do LAL são cirurgia de revisão, exame físico com *pivot shift* graus 2 ou 3, participação em esportes com mecanismo de *pivot* e/ou altos níveis de atividade, lassidão ligamentar e presença de fratura de Segond. Indicações secundárias podem incluir aqueles com idade abaixo de 25 anos, lesão crônica do LCA e sinais radiológicos de depressão do côndilo femoral lateral.

## Limitações

Nossa pesquisa incluiu apenas artigos publicados em inglês, com possível negligência de estudos de alta qualidade em outros idiomas. Além disso, a coleta de dados foi limitada ao banco de dados Scopus por ser conhecido por sua precisão, ampla cobertura e permitir a exportação de dados de citação; outros bancos de dados, como PubMed ou Cochrane Library, não foram incluídos em nossa busca. Além disso, nossa busca foi restrita ao ligamento especificamente descrito como “ligamento anterolateral”, o que pode ter excluído estudos relevantes que se referem a ele por outros nomes, como “ligamento capsular lateral” ou “banda oblíqua anterior”.

Outra limitação surge da natureza da pesquisa bibliométrica em si, pois os artigos incluídos podem não representar totalmente o padrão atual de cuidado e evidência. Apesar disso, a análise bibliométrica dos 100 artigos mais citados sobre o LAL oferece uma base valiosa para identificar tendências de pesquisa e destacar os principais centros acadêmicos. Essa análise também fornece uma lista com curadoria de artigos influentes, servindo como material de leitura essencial para pesquisadores e novos residentes que buscam desenvolver o corpo de literatura existente nesse tópico.

## Conclusão

A presente análise revela que a pesquisa sobre o LAL está crescendo, com contribuições significativas em anatomia e biomecânica. No entanto, estudos adicionais são necessários para estabelecer as melhores indicações para a reconstrução e as técnicas cirúrgicas ideais.
